# Breached Barriers: A Scoping Review of Blood-Central Nervous System Barrier Pathology in Amyotrophic Lateral Sclerosis

**DOI:** 10.3389/fncel.2022.851563

**Published:** 2022-03-31

**Authors:** Ario Mirian, Alexander Moszczynski, Serena Soleimani, Isabelle Aubert, Lorne Zinman, Agessandro Abrahao

**Affiliations:** ^1^Clinical Neurological Sciences, Western University, London Health Sciences, London, ON, Canada; ^2^School of Medicine, Queen's University, Kingston, ON, Canada; ^3^College of Osteopathic Medicine, Michigan State University, East Lansing, MI, United States; ^4^Biological Sciences, Hurvitz Brain Sciences Research Program, Sunnybrook Research Institute, Sunnybrook Health Sciences Centre, Toronto, ON, Canada; ^5^Department of Laboratory Medicine and Pathobiology, Temerty Faculty of Medicine, University of Toronto, Toronto, ON, Canada; ^6^Division of Neurology, Department of Medicine, Temerty Faculty of Medicine, University of Toronto, Toronto, ON, Canada; ^7^Division of Neurology, Department of Medicine, Sunnybrook Health Science Centre, Toronto, ON, Canada; ^8^Evaluative Clinical Sciences, Hurvitz Brain Sciences Research Program, Sunnybrook Research Institute, Sunnybrook Health Sciences Centre, Toronto, ON, Canada; ^9^Harquail Centre for Neuromodulation, Sunnybrook Research Institute, Toronto, ON, Canada

**Keywords:** blood-brain barrier, blood-spinal cord barrier (BSCB), motor neuron disease (MND), amyotrophic lateral sclerosis, neurovascular unit, pericyte

## Abstract

**Introduction:**

Recent studies have implicated changes in the blood-central nervous system barriers (BCNSB) in amyotrophic lateral sclerosis (ALS). The objective of this scoping review is to synthesize the current evidence for BCNSB structure and functional abnormalities in ALS studies and propose how BCNSB pathology may impact therapeutic development.

**Methods:**

A literature search was conducted using Ovid Medline, EMBASE, and Web of Science, from inception to November 2021 and limited to entries in English language. Simplified search strategy included the terms ALS/motor neuron disease and [BCNSB or blood-brain barrier (BBB) or blood-spinal cord barrier (BSCB)]. Henceforth, BCNSB is used as a term that is inclusive of the BBB and BSCB. Four independent reviewers conducted a title and abstract screening, hand-searched the reference lists of review papers, and performed a full text review of eligible studies. Included studies were original peer-reviewed full text publications, evaluating the structure and function of the BCNSB in preclinical models of ALS, clinical ALS, or postmortem human ALS tissue. There was no restriction on study design. The four reviewers independently extracted the data.

**Results:**

The search retrieved 2,221 non-duplicated articles and 48 original studies were included in the synthesis. There was evidence that the integrity of the BCNSB is disrupted throughout the course of the disease in rodent models, beginning prior to symptom onset and detectable neurodegeneration. Increased permeability, pharmacoresistance with upregulated efflux transporters, and morphological changes in the supporting cells of the BCNSB, including pericytes, astrocytes, and endothelial cells were observed in animal models. BCNSB abnormalities were also demonstrated in postmortem studies of ALS patients. Therapeutic interventions targeting BCNSB dysfunction were associated with improved motor neuron survival in animal models of ALS.

**Conclusion:**

BCNSB structural and functional abnormalities are likely implicated in ALS pathophysiology and may occur upstream to neurodegeneration. Promising therapeutic strategies targeting BCNSB dysfunction have been tested in animals and can be translated into ALS clinical trials.

## Introduction

Amyotrophic lateral sclerosis (ALS) is a terminal neurodegenerative disease that results in progressive wasting and paralysis of voluntary muscles. Only two medications, riluzole and edaravone, are approved with a marginal effect of slowing disease progression. ALS pathophysiology remains poorly understood, but multiple preclinical and postmortem studies, along with *in vivo* neuroimaging, and serum, CSF, and neurophysiological biomarkers, have been utilized to investigate this complex disease. These studies have demonstrated that ALS pathophysiology includes: hyperexcitability and degeneration of the motor network in the motor cortex, brainstem, and spinal cord; protein misfolding; impaired astrocytic and microglial functions; neuroinflammation; free radical toxicity; and mitochondrial and RNA metabolism dysfunction (Taylor et al., 2016; Eisen, 2021).

Studies led by Garbuzova-Davis et al. have implicated changes in the neurovascular unit in ALS pathology (Garbuzova-Davis et al., 2007a,b, 2012). The neurovascular unit comprises the capillary and surrounding neurons, astrocyte end-feet, microglia, and pericytes. In contrast to fenestrated and sinusoidal capillaries in other organs, the CNS capillaries are continuous and composed of a protective layer of tightly joined endothelial cells and basal membrane, known as the blood-brain barrier (BBB) and blood-spinal cord barrier (BSCB), hereafter collectively referred to as blood-central nervous system barriers (BCNSB) (Supplementary Figure 1).

The objective of this scoping review is to synthesize the current evidence for BCNSB structure and functional abnormalities described in ALS animal and human studies and propose mechanisms by which BCNSB-associated pathology may impact therapeutic development.

## Methods

### Literature Search

Literature search was conducted in November 2021 on the Ovid MEDLINE, EMBASE, and Web of Science databases for entries in English from inception to search date. Briefly, the search strategy (Supplementary Table 1) used the keywords: (“motor neuron disease” or “motor neurone disease” or “amyotrophic lateral sclerosis” or “ALS” or “primary lateral sclerosis” or “PLS” or “progressive muscle atrophy” or “PMA” or “Lou Gehrig Disease”) AND (“blood brain barrier” or “BBB” or “blood spinal cord barrier” or “BSCB” or “blood cerebral spinal fluid barrier” or “blood cerebrospinal fluid barrier” or “BCSFB” or “blood central nervous system barrier” or “BCNSB”).

### Eligibility Criteria, Data Extraction, and Synthesis

Four independent reviewers (Abrahao, Mirian, Moszczynski, and Soleimani) performed a primary title and abstract screening and extracted full data from eligible articles following the PRISMA extension for scoping reviews (PRISMA-ScR) guidelines (Tricco et al., 2018) and a pre-specified protocol registered with PROSPERO (CRD42017065405) to mitigate selection bias.

Included studies were original, peer-reviewed, full-text published or accepted articles assessing the following research questions: (1) in the context of ALS pathogenesis, what is known from the literature about the structure and function of the BBB and BSCB, as well as therapeutic interventions targeting these barriers, in animal and human ALS studies? (2) in the context of ALS treatment development, how does the BBB and BSCB impact therapeutic access to the CNS in animal and human ALS studies? Exclusion criteria included non-peer-reviewed articles; publications in the format of poster abstracts, editorial letters, conference papers; and articles without extractable data on ALS or its variants, primary lateral sclerosis (PLS) or progressive muscular atrophy (PMA). Studies cited in peer-reviewed review papers were also hand-searched for broader inclusion. Additional studies that fell outside the scope of the search strategy but were considered relevant were also included for the discussion of the clinical context of the BCNSB pathological findings and therapeutic delivery.

Data charting from each study was performed in duplicate by independent reviewers. Data items included the studied structure (BSCB and BBB), the research questions, interventions, experimental groups or population, sample size, animal disease stage (pre vs. symptomatic), ALS model description, BCNSB functional or structural measures, and descriptive findings. The synthesis and interpretation of the extracted data were presented in a descriptive manner without statistical inferences or meta-analyses given the largely qualitative nature of the studies in this review. Discrepancies in data extraction and synthesis were resolved by consensus decision of all reviewers. Supplementary Table 2 reports the PRISMA-ScR checklist.

## Results

The search yielded 2,221 non-duplicated entries. After the primary and full-text screening, data from 48 studies investigating BCNSB pathology in ALS were extracted (Supplementary Figure 2). Tables 1–3 summarize these studies according to preclinical, postmortem tissue, and clinical evidence, respectively.

**Table 1:** Preclinical studies investigating BCNSB integrity and function in ALS.

**References**	**Topography (BSCB, BBB or both)**	**Research question**	**Model**	**Sample size**	**Pre vs. symptomatic (preclinical models)**	**Direct/** **indirect measure of BCNSB**	**Measure**	**Findings**
Andjus et al. (2009)	BBB	Can BBB deterioration be detected by 7T MRI in mSOD1 rats?	mSOD1 (G93A) rats	*n* = 5 SOD1, *n* = 2 WT	Both	Direct	7T MRI	Presence of the contrast in brain tissue indicting BBB permeability.
Bataveljic et al. (2011)	BBB	To investigate inflammatory markers of disease using neuroimaging in mSOD1 rats	mSOD1 (G93A) rats	*n* = 5–12/group	Symptomatic	Indirect	1.5 T MRI	Gadolinium leakage through BBB occurs in areas of T cell infiltration.
Bataveljic et al. (2012)	BBB	Are AQP4 and Kir4 modified in a rodent model of ALS?	mSOD1 (G93A) rats	*n* = 3/group	Symptomatic	Direct	IHC, Western blot, patch-clamp	AQP1 and Kir4.1 coexpress and colocalize in astroglial endfeet lining the BBB. Upregulation of AQP1 in mSOD1 mice while Kir4.1 is downregulated.
Boston-Howes et al. (2008)	BSCB	Does the glutamate uptake enhancer NDGA prolong life in mSOD-1 mice?	mSOD1 (G93A) mice	*n* = 17	Both	Direct	Western Blot	Increases in P-gp expression over disease progression. Correlates with decrease in NDGA effect.
Boswell et al. (2013)	BSCB	Is there evidence of perfusion alteration in mSOD-1 mice?	mSOD1 (G93A) mice	Not stated	Symptomatic	Indirect	Radiotracers	IgG1 and (86)Rb crossed BSCB in SOD1(G93A) mice.
Chan et al. (2017)	BBB and BSCB	Is P-gp modified in mSOD-1 mice and what are the implications for therapeutics in ALS?	mSOD1 (G93A) rats	*n* = 4/group	Both	Direct	IHC, Western Blot	Activity and expression of P-gp significantly increases after symptom-onset in both BSCB and BBB. NFkB (increases P-gp) has no changes in nuclear localization on capillaries
Evans et al. (2014)	BBB	Can T2 weighted MRI detect pathological changes in mSOD-1 mice?	mSOD1 (G93A) mice	*n* = 10 behavioral, 4 MRI	Both	Indirect	7T MRI, Rotarod, IHC	No changes in vascular permeability, or endothelial activation were found at any stage of disease. No BBB breakdown or upregulation of endothelial VCAM-1 expression.
Eve et al. (2018)	BSCB	Does IV transplantation of human bone marrow CD34+ (hBM34+) cells in symptomatic ALS mice protect capillary integrity?	mSOD1 (G93A)mice	*n* = 6–9/group	Symptomatic	Direct	Light microscopy	Microhemorrhage incidence in spinal cord decreased in a dose-dependent manner with the injection of hBM34+ cells.
Garbuzova-Davis et al. (2007a)	BBB and BSCB	Is there evidence of BBB and BSCB dysfunction in SOD1 mice?	mSOD1 (G93A) mice	*n* = 3=8/group	Symptomatic	Direct	Electron microscopy	Vacuolation of endothelial cells. Layers of endothelium were degenerated, duplicated layers of BM. Edema in EC space, swollen astrocyte foot processes.
Garbuzova-Davis et al. (2007b)	BSCB	Is there evidence of BSCB compromise in mSOD1 mice?	mSOD1 (G93A) mice	*n* = 6–14/group	Symptomatic	Direct	Nissl staining, Immunofluorescence	Vessel permeability in early and late timepoints accompanied by pathological changes.
Garbuzova-Davis et al. (2017)	BSCB	Is endothelial repair an effective therapeutic in a mouse model of ALS?	mSOD1 (G93a) mice	*n* = 15–20/group	Symptomatic	NA	IHC, behavioral assessment	Neurobehavioral improvement 4 weeks post-treatment with human bone marrow CD34+ (hBM34+) cells.
Garbuzova-Davis et al. (2018)	BBB	Can human bone marrow stem cell transplantation repair BBB damage?	mSOD1(G93A) mice	*n* = 16–23/group	Symptomatic	Direct	Electron microscopy, IHC, Evans blue dye	Improved ultrastructural capillary morphology, capillary density, basement membrane integrity, axonal myelin coherence. Decreased BBB leakage.
Garbuzova-Davis et al. (2019)	BBB	Establish the effects of hBM-EPCs transplanted in mSOD1 mice at symptomatic disease stage	mSOD1(G93A) mice	*n* = 19–30/group	Symptomatic	Direct	Electron microscopy, IHC, Evans blue dye	Improved behavioral outcomes, capillary ultrastructure, perivascular astrocytic end feet, motor neuron survival. Decreased BBB permeability.
Garbuzova-Davis et al. (2021)	BSCB	Can marrow derived stem cells improve tight junction protein levels, and other BSCB measures in spinal cord of G93A SOD1 mutant mice?	mSOD1(G93A) mice	*n* = 15–17/group	Symptomatic	Direct	Western blot, IHC, fluorescent microscopy	Increased tight junction protein levels, capillary pericyte coverage, basement membrane laminin immunoexpression, and endothelial cytoskeletal F-actin fluorescent expressions.
Jablonski et al. (2014)	BSCB	Does improving riluzole CNS bioavailability through inhibition of P-gp and BCRP efflux transporters improve riluzole's therapeutic effects in mSOD1 mice	mSOD1 (G93A) mice	*n* = 5–6/group	Symptomatic	Indirect	Grip strength, mass spec, IHC, immunofluorescence	Human spinal cord tissue showed increased P-gp levels. In mice, riluzole administration in conjunction with P-gp/BCRP inhibitor elacridar improved survival and motor neuron count.
Lewandowski et al. (2016)	BSCB	Does PDGF-CC-induced BSCB dysfunction occur in ALS and might it modify disease course?	mSOD1 (G93A) mice with PDGFC inhibited or Knock-out, sALS	*n* = 4–12 ALS; *n* = 3–32 mice	Both	Indirect	IHC, Western blot	Increased expression of PDGFC and PLAT in sALS. Presymptomatic activation of the PDGF-CC pathway in mice. Decrease of Pdgfc expression in mice slowed progression of phenotype.
Meister et al. (2015)	BBB	Can mitant SOD1 impact tight junction stability and affect BBB integrity in an ALS model?	mSOD1 (G93A) mice	*n* = 3–6/group	Symptomatic	Direct	Western Blot, Radiotracers, immunohistochemistry	Reduced claudin-5 levels and a decreased transendothelial resistance (TER). Increased permeability to inulin in cells from SOD1-G93A mice. Repression of the claudin-5 gene expression in hSOD1(G93A) cells.
Miyazaki et al. (2011)	BBB	Evaluate changes in perivascular components and basement membrane in mSOD1 mice and ALS tissue	mSOD1 mice, sALS	*n* =3 ALS/*n* =3 ctrl	Both	Direct	IHC, western blot	Diameter and density of PCAM- capillary declined in presymptomatic stage. Collagen IV progressively declined.
								MMP-9 activity increased progressively. In the human tissue, evidence of BBB disruption.
Nicaise et al. (2009a)	BBB and BSCB	Is there evidence of BSCB and BBB impairment in mSOD-1 rats?	mSOD1 (G93A) rats	*n* = 4–8 per group	Both	Direct	IHC, PCR, EM	BSCB permeability increased in symptomatic rats only. BSCB pathology (IgG, hemosiderin) present in presymptomatic rats. Ocln and ZO-1 expression decreased in mSOD-1 rats.
Nicaise et al. (2009b)	BSCB	What is the effect of mSOD1 on AQP4 expression in a rat model?	mSOD1 (G93A) rats	*n* = 3–11/group	Symptomatic	Direct	IHC, immunofluorescence, Western blot, RT-PCR	AQP4 immunolabeling present around motor neurons.
Peake et al. (2017)	BBB	Can chemotherapeutic agents increase proliferation of bone marrow derived cells in the CNS of mSOD1 mice?	mSOD1 (G93A)mice	*n* = 3/group	Symptomatic	NA	Immunofluorescence	mSOD mice had greatest accumulation of BMDC cells with different morphology and distribution. GCSF does not increase BMDCs in CNS.
Qosa et al. (2016)	BSCB	Evaluate P-gp expression profile in spinal cord of SOD1 mice and potential role of mutation-bearing astrocytes in regulating P-gp.	mSOD1 (G93A) mice	*n* = 3 or more/group	Both	Direct	immunohistochemistry, western blot, activity assay	P-gp upregulation via ROS increase restricted to endothelial cells of the capillaries driven by mSOD1 astrocytes. Astrocytes expressing FUS-H517Q also drove upregulation of P-gp via TNF-α release.
Rabinovich-Nikitin et al. (2016)	BSCB	Investigate the effect on survival during chronic administration of small molecule AMD3100 to mSOD1 mice	mSOD1 (G93A) mice	*n* = 5/group	Pre-symptomatic	Direct	Evans blue	Decreased Evans blue and hemosiderin staining, along with increased tight junction marker levels (ZO-1, claudin 5) in mSOD1 rats that received BCNSB protective agent which was accompanied by increased survival.
Stamenković et al. (2017)	BBB	BBB permeability and the brain tissue redox status of the mSOD1 rats investigated by *in vivo* EPR spectroscopy.	mSOD1 (G93A) rats	*n* = 6/group	Both	Direct	EPR spectroscopy	Altered brain tissue redox status, and possibly BBB disruption in these animals.
Tang et al. (2021)	BSCB	Investigate endothelial barrier integrity and Ocln expression in mSOD-1 mice	mSOD1 (G93A) mice	*n* = 3/group	Presymptomatic	Direct	IHC, Western blot	mSOD-1 disrupted endothelial barrier integrity and downregulated Ocln expression with disease progression.
Watanabe-Matsumoto et al. (2018)	BBB	To investigate the expression of aquaporin 4 in a mouse model of ALS and in ALS patient tissue	mSOD1 (G93A) mice, LoxSOD1 (G37R) mice, AQP4 KO mice	*n* = 3/group	Both	Indirect	IHC and western blot	AQP4 is overexpressed in ALS models. Improvement in BBB permeability was observed in the AQP4-deficient ALS mice. Time to disease onset and lifespan were reduced in the AQP4-deficient ALS mice.
Winkler et al. (2014)	BSCB	Does BSCB damage contribute to motor neuron degeneration?	mSOD1 (G93A) mice	*n* = 14–21/group	Both	Direct	IHC, immunofluorescence	Warfarin-induced BSCB damage increased motor neuron damage. Reversal of BSCB damage increased motor neuron survival.
Zhong et al. (2008)	BBB	Does mutant SOD1 disrupt the BBB in mouse models of ALS?	mSOD1 (G93A) mice	3–6/group	Symptomatic	Direct	EM, qRT-PCR, IHC	BSCB changes occurred before motor neuron loss or symptoms. IgG staining from blood vessels in lumbar cord of dismutase-active SOD1. Hemosiderin outside of motor neurons in presymptomatic. Zo-1, Ocln, and claudin-5 were reduced.
Milane et al. (2010)	BBB	Investigate expression and function of P-gp and BCRP mSOD1 mice.	mSOD1 (G86R) mice	*n* = 6/group	Presymptomatic	Direct	RT-PCR, Western blot	Increased P-gp expression and function in presymptomatic mice. Riluzole brain disposition was decreased. BCRP expression and function unaltered.
Sasaki et al. (2015)	BSCB	To investigate the impact of motor neuron TDP-43 in BSCB integrity.	TDP-43 knockout mice and WT mice	3 per group	Pre and post	Direct	EM, light microscopy, Western blot	Altered endothelia, increased fibrinogen in early symptomatic stages. Resolved in late stage. Preserved tight junctions.
Ouali Alami et al. (2020)	BSCB	Can genetic modification of astrocytes function improve BSCB impairment in a mouse model of ALS?	SOD1, TDP-43, FUS, Tbk1 ALS mice	*n* = 5–8/group	Both	Direct	IHC, western blot	All models demonstrate impaired BSCB by all measures. DREDD modification of astrocytes to enhance MN firing improves BSCB integrity while inactivation of MN firing exacerbates it.
Jablonski et al. (2012)	BSCB	Does ALS drive increased expression of drug efflux transporters?	mSOD1 (G93A) and TDP43 (A315T) mice, 2 sALS and 1 fALS	*n* = 3/group	Both	Direct	Western Blot, RNA extraction, qRT-PCR	P-gp and BCRP increased in activity and expression with disease progression in mice. P-gp and BCRP protein expression also increased in spinal cords of ALS tissue.
Garbuzova-Davis et al. (2020)	BBB	Characterize EVs derived from hBM-EPCs as potential cell-free therapeutics for endothelium repair in ALS.	*In vitro* mouse brain	NA	NA	Indirect	Cell culture	EV uptake by cells and reduced mBEC damage from the pathological environment.
Mohamed et al. (2019)	BBB	Test the impact of glutamate excretion on P-gp expression in endothelial cells	*In vitro* ALS-derived astrocytes	NA	NA	Indirect	Western blot, ICC	Co-culture of endothelial cells with ALS-derived astrocytes increased P-gp expression levels and activity. NMDAR antagonism reduced this effect.

*hBM-EPCs, Human bone marrow-derived endothelial progenitor cells; EVs, extracellular vesicles; Hcy, homocysteine; BBB, blood-brain barrier; BCNSB, blood-CNS barrier; BSCB, blood-spinal cord barrier; CSF, cerebrospinal fluid; Hb, hemoglobin; TDP-43, TAR-DNA binding protein of 43 kDa; IHC, Immunohistochemistry; EM, electron microscopy; IgG, immunoglobulin; BNB, blood-nerve barrier; MVD, microvascular disease; ZO-1, tight junction protein-1; Ocln, occluding; fALS, familial ALS; sALS, sporadic ALS; RT-qPCR, real time quantitative polymerized chain reaction; CP, choroid plexus; MCSF, macrophage colony stimulating factor; VCAM-1, vascular cell adhesion molecule 1; VEGF, vascular endothelial growth factor; P-gp, P-glycoprotein; BCRP, breast cancer resistance protein; SC, spinal cord; mSOD1, mutant superoxide dismutase 1; NDGA, Nordihydroguaiaretic acid; FUS, fused in sarcoma; Tbk1, TANK binding kinase-1; TNF-a, tumor necrosis factor a; ROS, reactive oxygen species; PDGFC, platelet derived growth factor C; PLAT, plasminogen activator tissue type; MMP-9, matric metalloproteinase 9; mBEC, mouse brain endothelial cell; hTDP-43, human TDP-43*.

**Table 2:** Human post-mortem studies investigating BCNSB integrity and function in ALS.

**References**	**Topography (BSCB, BBB, or both)**	**Research question**	**Experimental group**	**Sample size**	**Pre vs. symptomatic (preclinical models)**	**Direct/** **indirect measure of BCNSB**	**Measure**	**Findings**
Ferrer et al. (2021)	BSCB	Is abnormal TDP-43 pathology observable in spinal cord and frontal cortex blood vessels of patients with sALS/FTLD-TDP?	sALS, FTLD-TDP	14 ALS, 11 FTLD-TDP	NA	Indirect	IHC	In sALS spinal cord, TDP-43 Ser403–404 deposits adjacent to the lumen.
Ferrer et al. (2021)	BSCB	Is abnormal TDP-43 pathology observable in spinal cord and frontal cortex blood vessels of patients with sALS/FTLD-TDP?	sALS, FTLD-TDP	14 ALS, 11 FTLD-TDP	NA	Indirect	IHC	In sALS spinal cord, TDP-43 Ser403–404 deposits adjacent to the lumen.
Garbuzova-Davis et al. (2012)	BBB and BSCB	Is there evidence of BBB and BSCB deterioration in sALS postmortem tissue?	sALS	25 sALS, 18 ctrl	NA	Direct	EM and IHC	Endothelial cell damage and pericyte degeneration. Accumulation of perivascular collagen, and fibrin. Increased microvascular density. IgG microvascular leakage. Reduced tight junction and adhesion protein. Downregulations of ZO-1, Ocln, and claudin-5.
Henkel et al. (2009)	BSCB	Are tight junction proteins different in ALS?	sALS and fALS	4 fALS, 30 sALS, 16 ctrl	NA	Indirect	RNA extraction, qRT-PCR	ZO-1 and Ocln spinal cord mRNAs were decreased in ALS
Ono et al. (1998)	BSCB	Evaluate collagen integrity in the spinal cord of ALS patient tissue	sALS	*n* =10 per group	NA	Direct	Light and electron microscopy	Reduced capillary integrity and increased collagen fragmentation in ALS
Sasaki (2015)	BSCB	To investigate BSCB integrity in postmortem ALS spinal cord tissue	sALS	12 per group	NA	Direct	EM	Capillaries smaller diameter in ALS, basement membrane thickened, higher rate of endothelial and pericyte changes in ALS
Van Vliet et al. (2020)	BBB	Investigated the expression and cellular distribution of the ABC transporters P-gp BCRP in SC, motor cortex, and cerebellum in sALS and fAL	ALS	25 ALS, 14 ctrl	NA	Indirect	IHC	Higher P-gp expression in reactive astroglial cells in SC and motor cortex in ALS. BCRP expression was higher in glia in the SC and in blood vessels and glia in the motor cortex of ALS patients. No difference between sALS and fALS.
Winkler et al. (2013)	BSCB	Is BSCB disruption with erythrocyte extravasation and pericyte loss present in human ALS?	sALS, fALS	*n* = 8 sALS, 3 fALS	NA	Indirect	IHC, immunofluorescence	Increase in perivascular hemoglobin deposits in ALS. Parenchymal accumulation of plasma-derived IgG, fibrin and thrombin in ALS.
Yamadera et al. (2015)	BSCB	Investigate the integrity of the microvasculature in ALS spinal cord tissue	sALS	25 ALS, 6 ctrl	NA	Direct	IHC	Microvascular disease increased in ALS.

*hBM-EPCs, Human bone marrow-derived endothelial progenitor cells; EVs, extracellular vesicles; Hcy, homocysteine; BBB, blood-brain barrier; BCNSB, blood-CNS barrier; BSCB, blood-spinal cord barrier; CSF, cerebrospinal fluid; Hb, hemoglobin; TDP-43, TAR-DNA binding protein of 43 kDa; IHC, Immunohistochemistry; EM, electron microscopy; IgG, immunoglobulin; BNB, blood-nerve barrier; MVD, microvascular disease; ZO-1, tight junction protein-1; Ocln, occluding; fALS, familial ALS; sALS, sporadic ALS; RT-qPCR, real time quantitative polymerized chain reaction; CP, choroid plexus; MCSF, macrophage colony stimulating factor; VCAM-1, vascular cell adhesion molecule 1; VEGF, vascular endothelial growth factor; P-gp, P-glycoprotein; BCRP, breast cancer resistance protein; SC, spinal cord; mSOD1, mutant superoxide dismutase 1; NDGA, Nordihydroguaiaretic acid; FUS, fused in sarcoma; Tbk1, TANK binding kinase-1; TNF-a, tumor necrosis factor a; ROS, reactive oxygen species; PDGFC, platelet derived growth factor C; PLAT, plasminogen activator tissue type; MMP-9, matric metalloproteinase 9; mBEC, mouse brain endothelial cell; hTDP-43, human TDP-43*.

**Table 3:** Clinical studies investigating BCNSB integrity and function in ALS.

**References**	**Topography (BSCB,** **BBB, or** **both)**	**Research question**	**Experimental group**	**Sample size**	**Pre vs. symptomatic (preclinical models)**	**Direct/** **indirect measure of BCNSB**	**Measure**	**Findings**	**Mean ALS-FRS score**	**Mean disease duration (months)**
Garbuzova-Davis et al. (2010)	BSCB	To investigate the use of endothelial cells in blood smears as a marker of ALS	sALS	*n* = 6–13/group	NA	Indirect	Blood smear	Reduced circulating endothelial cells in ALS blood smears.	36.5	23.2
Prell et al. (2021)	BBB	Use the D50 progression model to assess clinical relevance of BBB dysfunction in ALS	sALS	160 ALS, 31 ALS mimics	NA	Indirect	CSF albumin levels	No correlation between disease progression and BBB function. Limb onset disease was associated with BBB disruption	36.6	15.7
Verstraete et al. (2010a,b)	BBB	Is there neuroimaging evidence of BBB compromise in living ALS patients?	sALS	12 ALS 12 ctrl	NA	Indirect	7T MRI	None of the ALS patients had cerebral microbleeds	39.5	14.3
Waters et al. (2021)	BSCB	Quantify BSCB breakdown, determine if BSCB breakdown displays the same pattern as motor neuron loss and TDP-43 proteinopathy.	sALS	236 ALS, 87 ctrl (clinical) 13 ALS, 5 ctrl (postmortem)	NA	Indirect	CSF analysis and IHC	Hb leakage in ALS spinal cord. Motor neuron loss and TDP-43 proteinopathy present. CSF Hb elevated in ALS.	Not reported	Not reported
Wu et al. (2020)	BBB	Investigate the relationship between concentration of Hcy and BBB integrity indicated by CSF/serum albumin ratio	sALS	31 ALS, 34 ctrl	NA	Indirect	CSF analysis	CSF Hcy was positively correlated with albumin ratio	38.3	18.6

*hBM-EPCs, Human bone marrow-derived endothelial progenitor cells; EVs, extracellular vesicles; Hcy, homocysteine; BBB, blood-brain barrier; BCNSB, blood-CNS barrier; BSCB, blood-spinal cord barrier; CSF, cerebrospinal fluid; Hb, hemoglobin; TDP-43, TAR-DNA binding protein of 43 kDa; IHC, Immunohistochemistry; EM, electron microscopy; IgG, immunoglobulin; BNB, blood-nerve barrier; MVD, microvascular disease; ZO-1, tight junction protein-1; Ocln, occluding; fALS, familial ALS; sALS, sporadic ALS; RT-qPCR, real time quantitative polymerized chain reaction; CP, choroid plexus; MCSF, macrophage colony stimulating factor; VCAM-1, vascular cell adhesion molecule 1; VEGF, vascular endothelial growth factor; P-gp, P-glycoprotein; BCRP, breast cancer resistance protein; SC, spinal cord; mSOD1, mutant superoxide dismutase 1; NDGA, Nordihydroguaiaretic acid; FUS, fused in sarcoma; Tbk1, TANK binding kinase-1; TNF-α, tumor necrosis factor α; ROS, reactive oxygen species; PDGFC, platelet derived growth factor C; PLAT, plasminogen activator tissue type; MMP-9, matric metalloproteinase 9; mBEC, mouse brain endothelial cell; hTDP-43, human TDP-43*.

### BCNSB Structure and Function in ALS Preclinical Models

In transgenic superoxide dismutase-1 (SOD1) rodent models of ALS, studies have demonstrated abnormal neurovascular unit and BCNSB ultrastructure (Garbuzova-Davis et al., 2007a,b) with upregulated pharmacoresistance mechanisms (Jablonski et al., 2012). Neurovascular unit changes include dysfunction of glia, endothelial cells, and pericytes, downregulation of tight junction expression, upregulation of drug efflux proteins, and increased paracellular permeability. TAR-DNA binding protein of 43 kDa (TDP-43), TANK-binding kinase 1 (TBK1), and Fused In Sarcoma (FUS) rodent models of ALS have also shown impairment of the BCNSB manifesting as ZO-1 adherens protein dysfunction, reduced vascular density, decreased expression of AQP4 and increased albumin levels in the CSF, among other changes (Jablonski et al., 2012; Sasaki et al., 2015; Ouali Alami et al., 2020).

**Figure 1:**
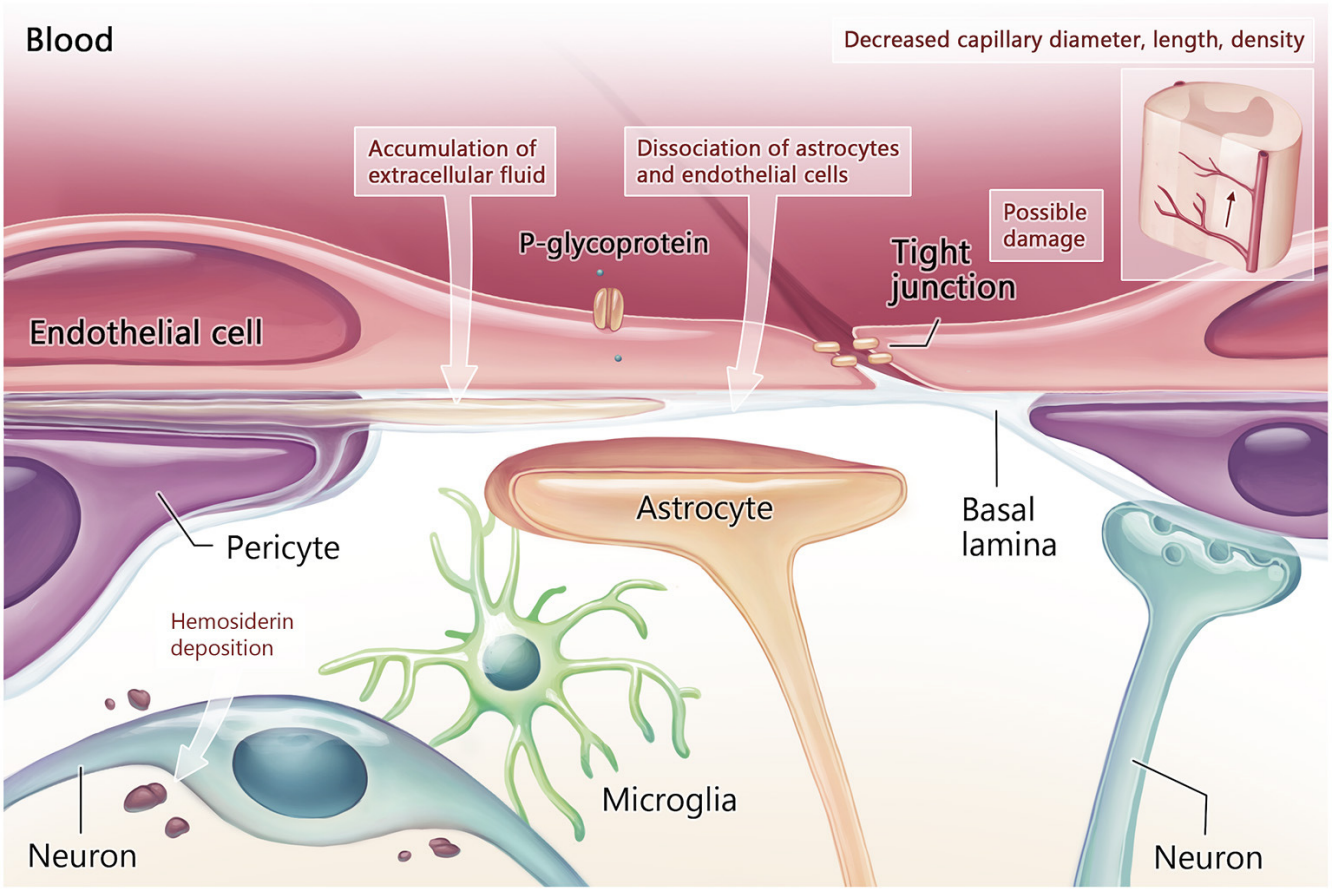
Blood-CNS barrier structure and function in the early stage of ALS. Early in the disease process, damage to tight junctions is associated with vascular pathology including reduction in capillary diameter, length, and density. Other structural damage includes dissociation of the astrocytes and endothelial cells of the neurovascular unit, which have been noted prior to motor neuron degeneration. Loss of neurovascular unit integrity leads to accumulation of extracellular fluid and hemosiderin deposition around motor neurons.

Early morphological changes in the BSCB preceded motor neuron degeneration in SOD1 rodent models, including reduced capillary density and premature dissociation of the astrocyte end-foot and endothelial cells (Figure 1; Zhong et al., 2008; Miyazaki et al., 2011). Blood flow was reduced by 30–45% in the lower spinal cord prior to symptom onset in SOD1 models compared to non-diseased animals (Zhong et al., 2008). During the presymptomatic stage, activation of the platelet-derived growth factor C (PDGF-CC) pathway may contribute to BSCB leakage (Lewandowski et al., 2016). Deposits of hemosiderin, a hemoglobin degradation marker and indicator of microhemorrhage, were observed in the anterior horn of SOD1 mice (Zhong et al., 2008) and SOD1 rats (Nicaise et al., 2009a). Through BSCB dysfunction, it was proposed that iron from hemoglobin metabolism, endogenous immune or inflammatory mediators (Garbuzova-Davis et al., 2019) and reactive oxidative stress can contribute to early motor neuron damage or exacerbate ongoing motor neuron damage (Winkler et al., 2014). Furthermore, electron microscopy demonstrated focal accumulation of extracellular fluid between vessel walls and adjacent parenchyma, along with swollen astrocyte foot processes in SOD1 rodents (Garbuzova-Davis et al., 2007a).

Ultrastructural abnormalities accumulated over time in the BSCB of symptomatic SOD1 animal models as neuronal loss progressed (Garbuzova-Davis et al., 2007a). Endothelial cells showed cytoplasmic disorganization, vacuolated mitochondria, and impaired basal membrane with reduced expression of tight junction proteins claudin-5, occludin, ZO-1 and proteoglycan agrin (Nicaise et al., 2009a; Garbuzova-Davis et al., 2012; Meister et al., 2015; Ouali Alami et al., 2020; Tang et al., 2021).

Structural changes were associated with progressive functional impairment of the BSCB in ALS animals. IgG, an immunoglobulin typically unable to cross the BSCB, had higher deposition in the extra- and intra-neuronal spaces within the spinal cord of pre-symptomatic and symptomatic SOD1 rodents (Zhong et al., 2008; Nicaise et al., 2009a; Garbuzova-Davis et al., 2012; Boswell et al., 2013; Rabinovich-Nikitin et al., 2016). The mechanism of IgG deposition remains unknown as it is unclear whether immunoglobulins have extravasated from the bloodstream through a disrupted BSCB, by transcytosis across endothelial cells (Poduslo et al., 1994), or have originated from a non-vascular mechanism of retrograde axonal transport by the motor neurons (Fabian and Petroff, 1987; Fratantoni et al., 1996). BSCB permeability increased with disease progression in SOD1 rodents, as suggested by the extravasation of intravenous Evans Blue dye into the spinal cord of symptomatic animals, in contrast to the lack of Evans Blue leakage in presymptomatic animals (Garbuzova-Davis et al., 2007a; Zhong et al., 2008; Nicaise et al., 2009b; Miyazaki et al., 2011).

Compared to the number of animal studies focusing on the spinal cord, fewer reports have demonstrated neurovascular unit changes in the brain and brainstem. BBB disruption in the midbrain of symptomatic SOD1 ALS rats was detected by the extravasation of gadolinium from the intravascular compartment into the midbrain on MRI. Gadolinium enhancement was correlated with activation of microglia and immune cell infiltration as detected by immunohistochemistry (Andjus et al., 2009; Bataveljic et al., 2011). Downregulation of potassium channel Kir4.1 and upregulation of water channel aquaporin-4 (AQP4) in astrocytic end-feet were associated with BBB dysfunction within the motor cortex and brainstem of mutant SOD1 rats (Bataveljic et al., 2012). This astrocyte-induced impairment of water and potassium homeostasis was indicated as the driving factor for increased BBB permeability and leakage of intravascular toxic mediators into the motor neuron microenvironment. Additional toxic effects occur with the accumulation of extracellular ions, such as potassium, which overcomes physiological buffer mechanisms in the ALS disease state (Bataveljic et al., 2012, 2014).

AQP4 upregulation has also been demonstrated in mouse models of ALS carrying the SOD1^G93A^, SOD1^G85R^, and LoxSOD1^G37R^ mutations (Watanabe-Matsumoto et al., 2018). In these studies, AQP4 mislocalization along astrocyte end-feet was unique to ALS pathology and was not seen in other nerve damage models, such as sciatic nerve axotomy. While these studies noted upregulation of AQP4, other studies have demonstrated downregulation (Ouali Alami et al., 2020). This discrepancy may be due to variability in models or timepoints, as the response of regulation of the protein may go through different phases of dysregulation after its equilibrium has shifted, downregulating and then upregulating to compensate or vice versa. Future research may focus on this phenomenon. Furthermore, SOD1 ALS mice with knocked out AQP4 gene did not develop BBB dysfunction to the same extent as their AQP4 expressing counterparts. Despite improved BBB integrity, SOD1 ALS mice lacking AQP4 demonstrated accelerated disease progression and shortened survival (Watanabe-Matsumoto et al., 2018). This seemingly opposite effect whereby a lack of AQP4 improved BBB integrity but worsened disease phenotype may be due to independent roles of AQP4 in BBB-mediated CNS homeostasis and ALS pathogenesis.

### Postmortem Evidence of BCNSB Dysfunction in ALS

**Figure 2:**
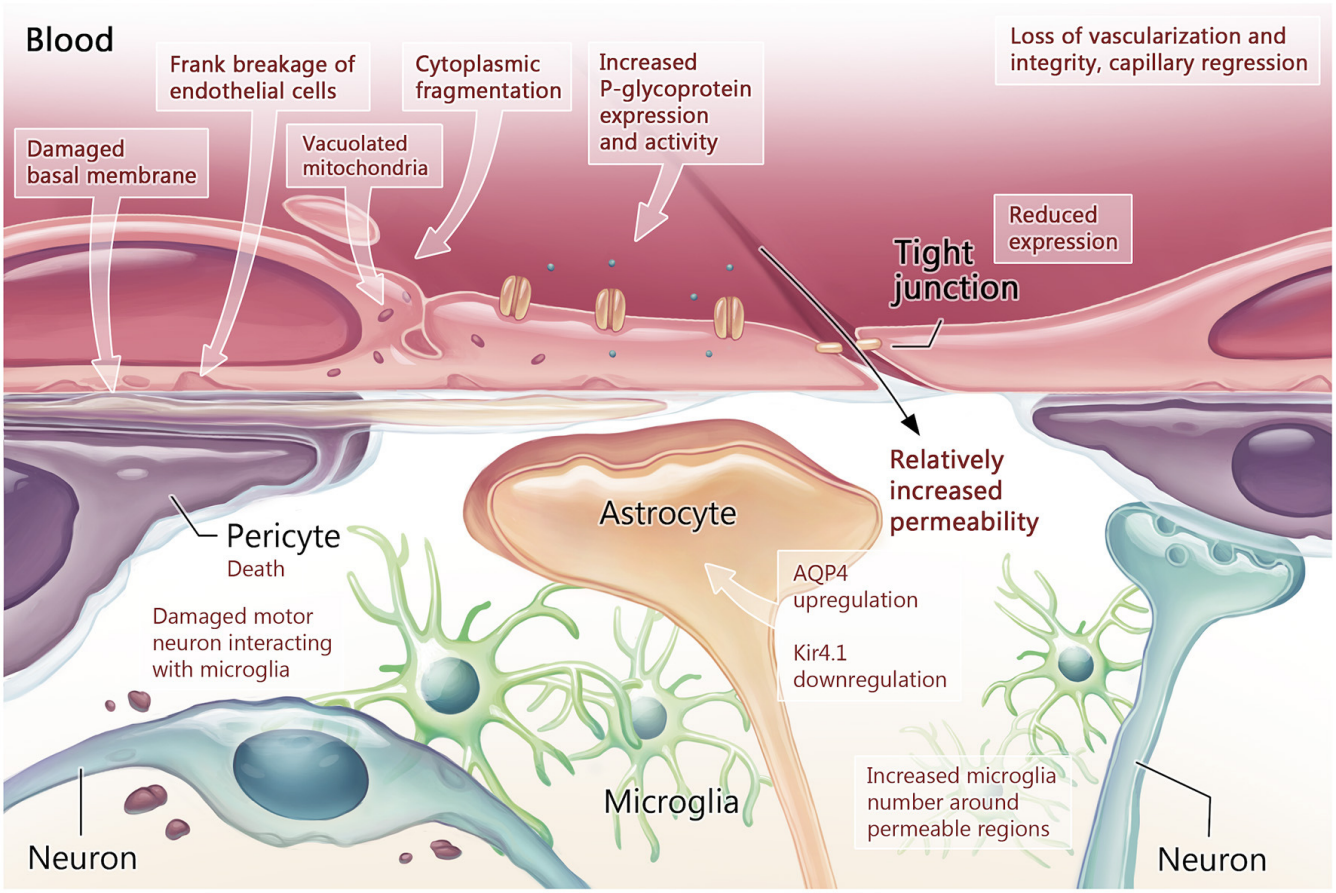
Blood-CNS barrier structure and function in the symptomatic stage of ALS. Once measurable behavioral changes have occurred, the early loss of neurovascular unit integrity has progressed to loss of vascularization through capillary regression. Tight junction protein expression is measurably reduced in endothelial cells, while export proteins such as P-glycoprotein (P-gp) demonstrate increased expression and activity. Within endothelial cells, signs of damage manifest as vacuolated mitochondria and cytoplasmic fragmentation. Damage to the basement membrane is also present. These changes culminate as breakage of the endothelial cell lining. With increased blood-CNS-barrier (BCNSB) permeability, changes in the cellular- molecular environment of the CNS can be measured including pericyte death and increased neuroinflammation as microglia satellite to damaged motor neurons and permeable regions. Astrocytes manifest molecular changes in the form of aquaporin 4 (AQP4) upregulation and potassium channel (Kir4.1) downregulation, leading to dysregulation the perineuronal environment.

**Figure 3:**
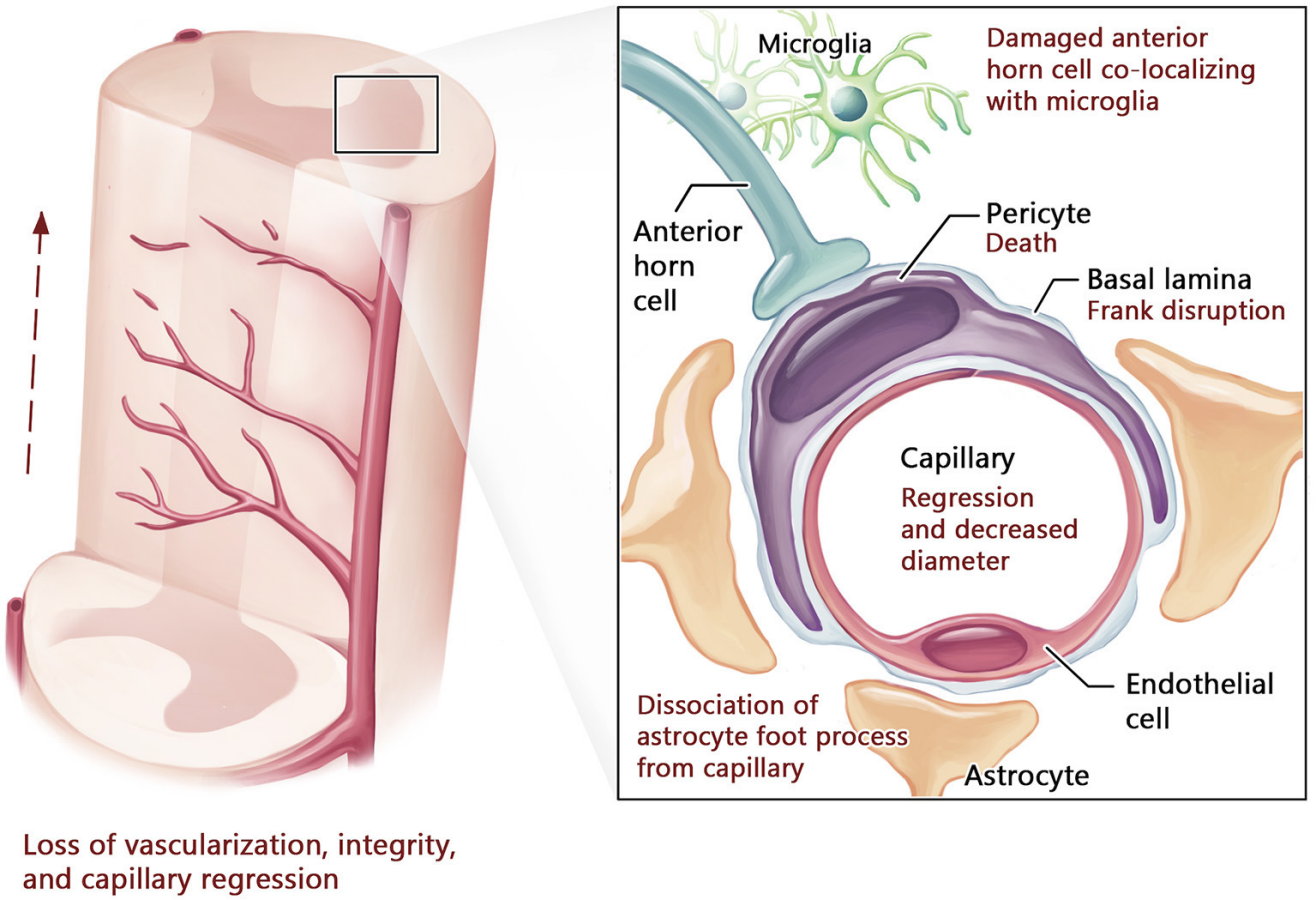
Blood-spinal cord barrier structure in advanced stage of ALS. In advanced ALS, neurovascular compromise progresses with further capillary regression and decreased diameter leading to reduced vascularization, particularly in the anterior horn of the spinal cord. This is accompanied by pericyte death, basal lamina disruption, collagen IV deposition, and marked dissociation of astrocyte foot processes from capillaries. Damaged cells within the anterior horn co-localize with microglia, indicating neuroinflammation.

The BCNSB findings observed in animal studies have also been corroborated in postmortem tissue from ALS patients (Garbuzova-Davis and Sanberg, 2014). In comparison to non-ALS human controls, there was disorganization of the microvascular architecture in the anterior horn, along with reduced capillary diameter (Sasaki, 2015) and density (Yamadera et al., 2015). Pericytes and endothelial cells within the spinal cord and brainstem exhibited degeneration, along with dissociation of astrocyte end-feet (Garbuzova-Davis et al., 2012; Winkler et al., 2013; Sasaki, 2015) and downregulation of tight junction proteins (Henkel et al., 2009). Disruptions in the endothelial lining indicating BCNSB breakage were also identified, with deposition of IgG, fibrin, thrombin, hemoglobin, and erythrocytes in the anterior horn tissue samples of patients with ALS (Miyazaki et al., 2011; Winkler et al., 2013; Sasaki, 2015). In the interstitial component of the BSCB, there was evidence for reduced and fragmented collagen bundles in the anterior horn of patients with ALS (Ono et al., 1998; Garbuzova-Davis and Sanberg, 2014). There was significant accumulation of perivascular collagen IV in the spinal cord and medulla (Garbuzova-Davis et al., 2012), a factor that can limit effective drug influx to the CNS (Garbuzova-Davis et al., 2016). The morphological and functional changes in the BCNSB in the late stages of ALS are schematized in Figures 2, 3.

TDP-43 deposition in endothelial cells has been observed in both ALS and frontotemporal lobar dementia with TDP-43 pathology (FTLD-TDP) in postmortem tissue (Ferrer et al., 2021). This has led to speculation over whether the pathological protein is being taken up by endothelial cells from neurons, or whether the observed deposits are from endogenous TDP-43 in the endothelial cells themselves. Both cases indicate that a vasculopathy is present in TDP-43 proteinopathies.

Whether the post-mortem BCNSB changes described in the vicinity of lower motor neurons (LMNs) in the spinal cord and brainstem also apply to the BBB surrounding the upper motor neurons (UMNs) in primary motor cortex is undetermined. Historically, studies in ALS have primarily investigated the LMNs and their connectivity with muscles at the neuromuscular junction, with less focus on the motor cortex. Given the robust data supporting a key pathological role of UMN dysfunction in ALS (Eisen, 2021), future preclinical, clinical and post-mortem studies on the interplay of astrocytes, UMNs, and motor cortex capillaries are warranted.

### Clinical Evidence of BCNSB Dysfunction in Patients Living With ALS

This scoping review did not identify validated imaging, serum, or CSF biomarkers to measure BCNSB integrity and function directly and reliably in patients living with ALS. Therefore, our understanding of BCNSB pathology throughout early to late stages of ALS in humans is limited. However, a small number of studies attempted to evaluate the BCNSB function in patients with ALS using pharmacokinetic assumptions and indirect markers.

Among these indirect measures, increased cerebrospinal fluid (CSF) total protein or albumin levels and CSF/serum concentration ratio of any given therapeutic may correlate with BCNSB permeability. Elevated CSF protein has been reported in some case series of ALS (Chelstowska and Kuzma-Kozakiewicz, 2014). However, protein may also leak into the CSF from the degenerating motor roots, limiting the utility of this measure as a BCNSB functional biomarker. For instance, breakdown of the blood-peripheral nerve barrier has been demonstrated by gadolinium enhancement on MRI of lumbosacral roots and leptomeninges of patients with ALS (Luigetti et al., 2010; Young et al., 2010). Circulating endothelial cells in peripheral blood was not a reliable indicator of BCNSB endothelial damage in patients with ALS (Garbuzova-Davis et al., 2010).

Measuring BCNSB permeability using CSF/serum therapeutic concentrations has limitations. It relies on the assumption of therapeutic extravasation from the vascular compartment to the CNS parenchyma and then to the subarachnoid space without direct CSF excretion at the choroid plexus (i.e., via the BCSFB) or complete therapeutic metabolism within neural tissue. Pharmacokinetic studies investigating CSF/serum concentration ratios of multiple ALS therapeutics have typically demonstrated low CSF/serum ratios suggesting limited therapeutic access across the BCNSB (Sussmuth et al., 2010; Wu et al., 2020; Prell et al., 2021; Waters et al., 2021).

Current structural and functional neuroimaging techniques lack the spatial resolution and accuracy required to directly assess the structure and function of BCNSB in patients with ALS. Radiologically, the BCNSB appears intact in ALS patients on standard imaging techniques, in contrast to multiple sclerosis and other CNS inflammatory conditions whereby BCNSB disruption is typically demonstrated by gadolinium extravasation on MRI. While early clinical ALS studies using novel 7T MRI scanners have largely explored the CNS anatomy and connectivity (Verstraete et al., 2010b; Cosottini et al., 2016; Barry et al., 2021), BCNSB leakage has not been extensively investigated with this technique. Of note, indirect measures of BBB integrity such as microbleeds in ALS were not increased as measured by 7T MRI (Verstraete et al., 2010a).

### Implications for Therapeutic Development in ALS

Despite increased permeability in preclinical and postmortem models, therapeutics with high molecular weight still have limited access to the CNS (Garbuzova-Davis et al., 2016). While lipid soluble and low molecular weight molecules can diffuse across the endothelial cell membranes, most of these compounds do not effectively enter the CNS and are transported back to the bloodstream by BCNSB-driven efflux or pharmacoresistance systems.

These pharmacoresistance proteins include specialized ATP binding cassette (ABC) efflux transporters that actively pump a number of endogenous and exogenous substrates out of the BCNSB (Mohamed et al., 2017). Relevant to ALS, the upregulation of key pharmacoresistance efflux proteins, P-glycoprotein (P-gp) and breast cancer resistance protein (BCRP), has been shown in multiple animal models (Milane et al., 2010; Jablonski et al., 2012; Chan et al., 2017), a finding also supported by postmortem human tissue studies (Jablonski et al., 2012; Qosa et al., 2016; Van Vliet et al., 2020).

Increased expression and activity of P-gp began after symptom onset in the BSCB and BBB of mutated SOD1 models (Boston-Howes et al., 2008; Milane et al., 2010; Chan et al., 2017). TDP-43^A315T^ mutant mice also demonstrated P-gp and BCRP overexpression (Jablonski et al., 2012). *In vitro*, the P-gp upregulation in the endothelial cells was mediated by nuclear factor κB (NF-κB) activation and was induced when these cells were co-cultured with ALS-derived astrocytes (Qosa et al., 2016; Mohamed et al., 2019). In postmortem tissue, P-gp and BCRP upregulation was observed in the spinal cord and motor cortex of both sporadic and familial ALS cases (Van Vliet et al., 2020). It has been suggested that P-gp evolved for the purpose of responding to harmful substances (Broeks et al., 1995). Therefore, in ALS it may be responding to the toxic sequelae of aberrant protein in the CNS or other circulating toxins. Upregulation may be the result of a physiological attempt to dispense of harmful substrates. A consequence, however, may be decreased drug efficacy.

Of clinical relevance, riluzole, the first approved ALS drug, is a substrate of these efflux proteins (Milane et al., 2009b, 2010). The P-gp overexpression over the course of the disease may explain the poor bioavailability of riluzole within CNS parenchyma thereby limiting its therapeutic efficacy (Milane et al., 2010; Jablonski et al., 2014). In contrast, edavarone, a free radical scavenger approved for ALS treatment, has negligible P-gp binding and is potentially less influenced by BCNSB efflux activity (Dash et al., 2018; Hyung et al., 2018).

Reversing BCNSB pharmacoresistance in ALS is a long-sought approach to enhance therapeutic effect via inhibition of efflux transporters (Milane et al., 2009a; Jablonski et al., 2014). There was a significant increase in the parenchymal concentration of riluzole in rodents co-treated with efflux transporter inhibitors, such as minocycline (Milane et al., 2009a), verapamil liposomes (Yang et al., 2018) and elacridar (Jablonski et al., 2014). For instance, riluzole plus elacridar, a third-generation inhibitor BCRP and P-gp, improved muscle function, disease progression and survival in ALS mice (Jablonski et al., 2014).

While inhibition of efflux transporters is a promising strategy for clinical trials there are some challenges with current P-gp inhibitors. This includes the high dose needed to block P-gp which may lead to adverse reactions and systemic toxicity (Milane et al., 2007; Kalvass et al., 2013), as well as the need for selectivity and specificity of P-gp only at the BBB to prevent adverse effects on other organs (Amin, 2013).

### BCNSB Pathology as a Therapeutic Target to Ameliorate ALS Disease Progression

Repair of the BCNSB pathology in ALS via stem cell transplantation has provided some evidence of symptomatic improvement in SOD1^G93A^ mice. The intravenous transplantation of human bone marrow-derived CD34+ cells (hBM34+) and endothelial progenitor cells (hBM-EPCs) enhanced the replacement of damaged endothelial cells in the CNS capillaries (Garbuzova-Davis et al., 2017, 2018, 2019, 2021; Eve et al., 2018). One mechanism of hBM-EPCs on the endothelium includes excretion of extracellular vesicles that transfer biomolecules to facilitate the repair of damaged microvascular endothelium in ALS (Garbuzova-Davis et al., 2020). Additionally, mice receiving hBM-EPCs have increased tight junction protein levels, capillary pericyte coverage, and basement membrane laminin expression, all of which maintain capillary endothelium integrity (Garbuzova-Davis et al., 2021). Ultimately, these mechanisms may prevent the entry of immune or inflammatory mediators which can contribute to motor neuron dysfunction (Garbuzova-Davis et al., 2019). At the symptomatic disease stage, hBM-EPC-treated mice have shown an improvement of behavioral outcomes and motor neuron survival, making it a promising therapeutic strategy for future translational clinical trials (Garbuzova-Davis et al., 2019).

Other therapeutics, such as an antagonist of the chemokine receptor CXCR4 (Rabinovich-Nikitin et al., 2016) and the myelosuppressive dealkylating agent busulfan (Peake et al., 2017), demonstrated improved BCNSB function in ALS models. Edaravone has been previously shown to mediate BBB repair in models of ischemic stroke, in addition to its primary mechanism of action as a free radical scavenger (Miyamoto et al., 2014; Tóth et al., 2014; Watanabe et al., 2015). Yet, BCNSB repair largely remains only a theoretical consideration in edavarone's mechanism of action in ALS.

## Discussion

ALS pathophysiology remains poorly understood and has typically focused on the interplay between degenerating motor neurons and activated glia. Recent evidence supported a key role of neurovascular unit pathology contributing to motor neuron degeneration in ALS. In this scoping review, rodent models demonstrated progressive BCNSB disruption throughout the course of the disease, beginning prior to detectable motor neuron loss. Morphological changes in the supporting cells of the BCNSB, including pericytes, astrocytes, and endothelial cells, were observed in animal models and postmortem studies of ALS patients. Progressive BCNSB changes have also been described in other neurodegenerative disorders, such as Alzheimer's disease (Zipser et al., 2007) and Parkinson's disease (Pan and Nicolazzo, 2018), suggesting commonalities in vascular dysfunction and neurodegeneration in the context of different proteinopathies.

BCNSB leakage may be driven by activated astrocytes, imbalance of extracellular ion and water channel homeostasis (Watanabe-Matsumoto et al., 2018), and oxidative stress (Garbuzova-Davis et al., 2016). Although it remains unclear whether the neurovascular unit insult is the driving causative mechanism in ALS pathophysiology, the studies in this scoping review indicate that it is likely to contribute to motor neuron degeneration as an early or upstream mechanism. Disrupted barriers can allow endogenous immune or inflammatory mediators to enter the motor neuron microenvironment (Garbuzova-Davis et al., 2008, 2019). Activated astrocytes lose their ability to provide trophic and metabolic support and become toxic to motor neurons (Nagai et al., 2007; Haidet-Phillips et al., 2011).

Evidence of BCNSB abnormalities in animal models and human postmortem tissue need further *in vivo* investigation in patients with ALS. Part of the endothelial cell injury in SOD1 rodents relates to a direct toxic effect of the mutated SOD1 protein and oxidative stress (Garbuzova-Davis et al., 2016), which may not be translatable to the majority sporadic ALS cases. Progressive respiratory failure and aspiration pneumonia, common causes of death among patients (Corcia et al., 2008), along with disrupted blood-gas homeostasis and a systemic inflammatory response, may account for some of the BCNSB abnormalities in end-stage ALS. These factors may increase tissue susceptibility to anoxia from the time of death to histological fixation as compared to non-ALS controls. Novel non-invasive imaging, serum and CSF biomarkers are warranted to ascertain BCNSB changes throughout the stages of human ALS.

Despite the evidence of BCNSB leakage in ALS models, therapeutic access to the CNS remains a challenge as these barriers still limit the passage of most therapeutics (e.g., antibodies, proteins, gene carriers and cells) from the vascular compartment to the CNS parenchyma. Limited therapeutic access to the CNS may account, at least in part, for the discrepancies between large therapeutic effect sizes observed in SOD1 mouse trials compared to lack of benefit in ALS clinical trials (Garbuzova-Davis et al., 2016). An important distinction between mutant SOD1 animal models and sporadic ALS patients is the degree of pericyte degeneration and perivascular collagen-IV accumulation in the latter (Garbuzova-Davis et al., 2016). Pericyte degeneration has been shown to reduce capillary blood flow which in turn may limit therapeutic delivery (Garbuzova-Davis and Sanberg, 2014; Garbuzova-Davis et al., 2016). Collagen-IV build up in the brain and spinal cord vessels may be a compensatory mechanism to BCNSB dysfunction and can further limit the diffusion of therapeutics across the BCNSB (Garbuzova-Davis et al., 2012, 2016). In addition, the upregulation of transmembrane efflux transporters, such as P-gp, further reduces drug bioavailability to the motor neuron network. Contrasting to SOD1 mice, the scarceness of *in vivo* proof of BCNSB disruption in patients with ALS may reflect the human disease heterogeneity and the lack of optimal tools to elucidate BCNSB function in patients.

Multiple strategies to enhance therapeutic delivery to the brain and spinal cord across the BCNSB have been reported in ALS preclinical and clinical studies. In addition to safety, ideally, BCNSB-modifying techniques should be temporary and reversible to avoid worsening of chronic BCNSB pathology in ALS. These approaches included the co-administration of mannitol for increased BCNSB permeability (Chi et al., 2011) or elacridar for P-gp inhibition (Jablonski et al., 2014), and direct tissue injections via open surgery. In humans, interventions involving invasive injection of therapeutics into the motor cortex (Martínez et al., 2009) and spinal cord (Feldman et al., 2014) have been performed. However, in addition to uncertain efficacy, the generalizability of surgical approaches, particularly for repeated procedures, is limited due to morbidity and tolerability in ALS patients with respiratory impairment.

As a less invasive alternative to bypass the BCNSB, repeated intrathecal injections of antisense oligonucleotides targeting SOD1 (Miller et al., 2013, 2020), gene therapy with adeno-associated virus rh10 containing an anti-SOD1 microRNA (AAV-miR-SOD1) (Mueller et al., 2020), and mesenchymal stem cells (Petrou et al., 2016; Cudkowicz et al., 2021) have been safely tested in humans with ALS. Non-invasive magnetic resonance-guided focused ultrasound (MRgFUS) has emerged as a technique to open the BBB safely and temporarily for targeted drug delivery to the motor cortex in patients with ALS (Abrahao et al., 2019). MRgFUS combines transcranial acoustic energy and intravenous microbubbles to disrupt the tight junctions of the targeted capillaries, allowing large therapeutics such as antibodies to gain access to the human human brain (Meng et al., 2021). Also, reversible MRgFUS-induced BBB permeability was safely performed in patients with Alzheimer's disease (Lipsman et al., 2018; Rezai et al., 2020; Park et al., 2021), another neurodegenerative condition with chronic BCNSB leakage (Zipser et al., 2007).

While the search strategy employed broad terms, it is possible that some relevant studies were missed and thus not reflected here. This scoping review aimed to summarize qualitative and quantitative BCNSB measures from heterogenous ALS study models but did not attempt to conduct meta-analyses or a critical appraisal of individual studies. To mitigate bias, four independent reviewers analyzed the extracted data and achieved consensus on data synthesis. Therefore, despite all the efforts to mitigate selection bias, reporting bias is still a possibility. The generalizability of the findings is also limited given the majority of studies investigated BCNSB changes in mutated SOD1 animal models with a relative paucity of studies in other models of motor neuron disease (e.g., FUS or TDP-43) and clinical data. Lastly, this review did not focus on the BCSFB at the choroid plexus. Given the structural differences in relation to the BBB and BSCB and recent reports of BCSFB disruption (Saul et al., 2020) and potential therapeutic target in ALS (Kunis et al., 2015), a dedicated review on this topic is suggested.

This synthesis of the literature demonstrates evidence that BCNSB structural and functional abnormalities are likely implicated in ALS pathophysiology. These BCNSB changes in preclinical models may represent an upstream process in relation to motor neuron degeneration. Therefore, more studies are needed to validate these findings *in vivo* in humans with ALS and to further elucidate the potential role of BCNSB disruption as a component of the disease pathophysiology. Promising therapeutic strategies targeting these BCNSB changes in animal models can be translated into future ALS clinical trials.

## Data Availability Statement

The original contributions presented in the study are included in the article/Supplementary Material, further inquiries can be directed to the corresponding authors.

## Author Contributions

AMi, AMo, SS, IA, and LZ: acquisition of data, analysis and interpretation of data, and manuscript writing. AA: study conceptualization, acquisition of data, analysis and interpretation of data, and manuscript writing. All authors contributed to the article and approved the submitted version.

## Funding

This research was undertaken, in part, thanks to funding from the Canada Research Chairs Program (to IA; CRC Tier 1 in Brain Repair and Regeneration), ALS Society of Canada and Brain Canada (to AA and LZ), and generosity of philanthropic gifts to the Sunnybrook Foundation for ALS research.

## Conflict of Interest

The authors declare that the research was conducted in the absence of any commercial or financial relationships that could be construed as a potential conflict of interest.

## Publisher's Note

All claims expressed in this article are solely those of the authors and do not necessarily represent those of their affiliated organizations, or those of the publisher, the editors and the reviewers. Any product that may be evaluated in this article, or claim that may be made by its manufacturer, is not guaranteed or endorsed by the publisher.
