# Supplementary material for: Breached Barriers: A Scoping Review of Blood-Central Nervous System Barrier Pathology in Amyotrophic Lateral Sclerosis
Source: Front Cell Neurosci. 2022 Mar 31;16:851563. doi: 10.3389/fncel.2022.851563 (PMC9009245; doi:10.3389/fncel.2022.851563)
Supplement: Supplementary file 1 [file Image_1.PDF]

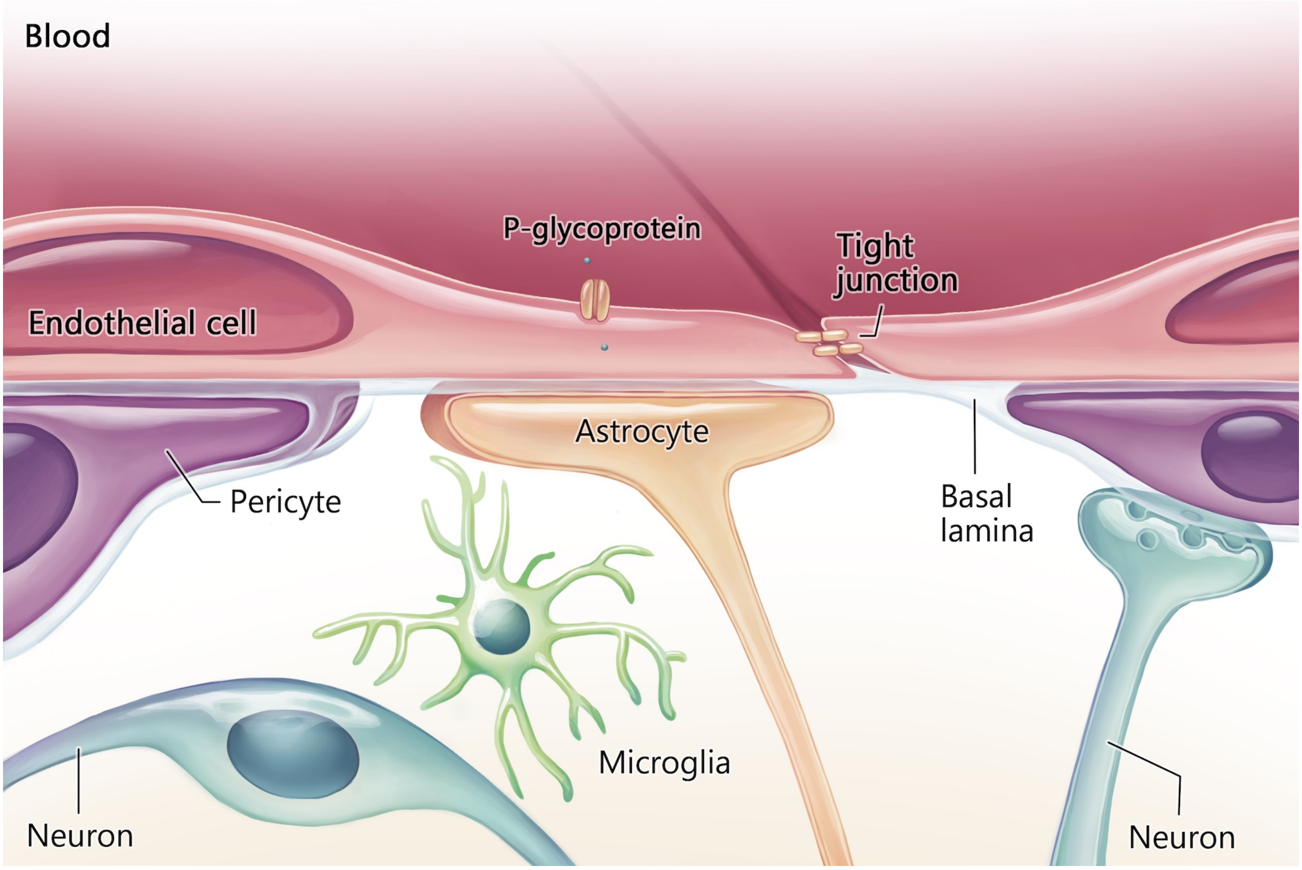

Supplementary Figure 1. Normal structure and function of blood-CNS barriers. The blood-CNS barriers (BCNSB) are composed of endothelial cells which work in conjunction with neurons, astrocytes, pericytes, and microglia to maintain the homeostasis of the central nervous system (Abbott et al., 2006;Fisher, 2009). Endothelial cells are adjoined by tight junctions which prevent passive diffusion of large molecules between cells, forcing trans-cellular molecular trafficking. The capillaries of the CNS are also associated with perivascular end-feet of astrocytic glia, pericytes, and microglia. Astrocytes contain ion channels which facilitate controlled molecular trafficking through the BCNSB. The BCNSB regulates blood flow, nutrient transport, ion homeostasis and the clearance of toxic substrates from the CNS. Small gaseous molecules and small lipophilic agents (less than 400 Da) can diffuse freely through lipid membrane (Grabrucker et al., 2016). Large hydrophilic molecules are transferred by specific receptor-mediated transcytosis or adsorptive-mediated transcytosis (Pardridge, 2003). There are also important efflux transporters expressed in the BCNSB, which eliminate substances from the CNS. These include P-glycoprotein (P-gp) and breast cancer resistance protein (BCRP).

References:

Abbott, N.J., Ronnback, L., and Hansson, E. (2006). Astrocyte-endothelial interactions at the blood-brain barrier. *Nat Rev Neurosci* 7**,** 41-53.

Fisher, M. (2009). Pericyte signaling in the neurovascular unit. *Stroke* 40**,** S13-15.

Grabrucker, A.M., Ruozi, B., Belletti, D., Pederzoli, F., Forni, F., Vandelli, M.A., and Tosi, G. (2016). Nanoparticle transport across the blood brain barrier. *Tissue Barriers* 4**,** e1153568.

Pardridge, W.M. (2003). Blood-brain barrier drug targeting: the future of brain drug development. *Mol Interv* 3**,** 90-105, 151.
