# Supplementary figures and images for: Breached Barriers: A Scoping Review of Blood-Central Nervous System Barrier Pathology in Amyotrophic Lateral Sclerosis
Source: Front Cell Neurosci. 2022 Mar 31;16:851563. doi: 10.3389/fncel.2022.851563 (PMC9009245; doi:10.3389/fncel.2022.851563)

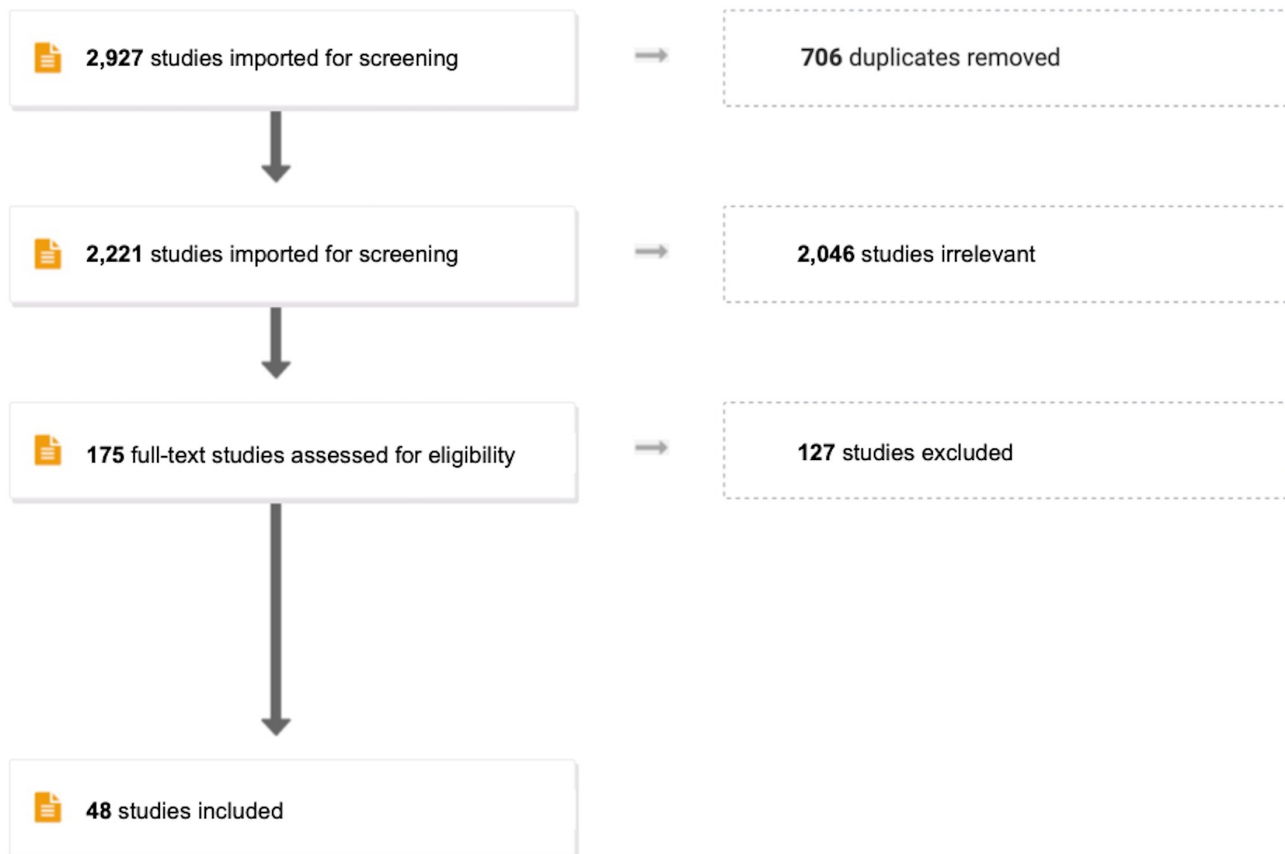

**Supplementary Figure 2. PRISMA flow diagram.**

Supplement: Supplementary file 2 [file Image_2.PDF]
