# Supplementary material for: Breached Barriers: A Scoping Review of Blood-Central Nervous System Barrier Pathology in Amyotrophic Lateral Sclerosis
Source: Front Cell Neurosci. 2022 Mar 31;16:851563. doi: 10.3389/fncel.2022.851563 (PMC9009245; doi:10.3389/fncel.2022.851563)
Supplement: Supplementary file 3 [file Table_1.DOCX]

Breached Barriers: A Scoping Review of Blood-Central Nervous System Barrier Pathology in Amyotrophic Lateral Sclerosis

Ario Mirian^1†^, Alexander Moszczynski^2†^, Serena Soleimani^3^, Isabelle Aubert^4,5^, Lorne Zinman^4,6,7^*, Agessandro Abrahao^4,6,7,8^*

Supplementary Material

**Supplementary Table 1: Search Strategy**

| **Database** | **Search Criteria** |
| --- | --- |
| Ovid MEDLINE(R) Epub Ahead of Print, In-Process & Other Non-Indexed Citations, Ovid MEDLINE(R) Daily and Ovid MEDLINE(R) from 1946 to November 2021 | 1 exp Blood-Brain Barrier/  2 blood brain barrier.mp.  3 blood spinal cord barrier.mp.  4 (CSF barrier or cerebral spinal fluid barrier or cerebrospinal fluid barrier).mp.  5 (CNS barrier or central nervous system barrier).mp.  6 or/1-5  7 exp Amyotrophic Lateral Sclerosis/  8 exp Motor Neuron Disease/  9 primary lateral sclerosis.mp.  10 progressive muscular atrophy.mp.  11 ALS.mp.  12 PLS.mp.  13 Lou Gehrig Disease.mp.  14 or/7-13  15 6 and 14  16 limit 15 to English language |
| Embase Classic + Embase from 1947 to November 2021 | 1 exp blood brain barrier/  2 exp blood cerebrospinal fluid barrier/  3 blood brain barrier.mp.  4 blood spinal cord barrier.mp.  5 (CSF barrier or cerebral spinal fluid barrier or cerebrospinal fluid barrier).mp.  6 (CNS barrier or central nervous system barrier).mp.  7 or/1-6  8 exp amyotrophic lateral sclerosis/  9 exp motor neuron disease/  10 exp progressive muscular atrophy/  11 exp primary lateral sclerosis/  12 spinal muscular atrophy.mp.  13 ALS.mp.  14 PLS.mp.  15 Lou Gehrig Disease.mp.  16 or/8-15  17 7 and 16  18 limit 17 to English language |
| Web of Science. Two sub-files searched: Science Citation Index Expanded (1900 – November 2021), Conference Proceedings Citation Index – Science (1990 – November 2021) | #1 TS=(blood brain barrier) OR TS=(blood spinal cord barrier) OR TS=(cerebral spinal fluid barrier) OR TS=(cerebrospinal fluid barrier) OR TS=(CSF barrier) OR TS=(central nervous system barrier) OR TS=(CNS barrier)  Indexes=SCI-EXPANDED, CPCI-S Timespan=All years  #2 TS=(amyotrophic lateral sclerosis) OR TS=(ALS) OR TS=(motor neuron* disease) OR TS=(Lou Gehrig disease) OR TS=(progressive muscular atrophy) OR TS=(spinal muscular atrophy) OR TS=(primary lateral sclerosis) OR TS=(PLS)  Indexes=SCI-EXPANDED, CPCI-S Timespan=All years  (#2 AND #1) *AND* **LANGUAGE:** (English)  Indexes=SCI-EXPANDED, CPCI-S Timespan=All years |
